# Lipid Metabolic Disorders in Neurodegenerative Diseases – Role of Androgen Receptor

**DOI:** 10.5152/eurasianjmed.2023.23024

**Published:** 2023-12-01

**Authors:** Satvika Sharma, Avneet Saini, D.K. Dhawan

**Affiliations:** Department of Biophysics, Panjab University, Chandigarh, India

**Keywords:** Androgen receptor, neurodegenerative diseases, lipid metabolism

## Abstract

The challenge of managing neurodegenerative disorders is a worldwide concern, especially in the aging population. Neurodegenerative diseases are a varied group of disorders that are characterized by progressive degeneration of the structure and function of the nerve cells. Neurodegenerative diseases are increasing at an alarming rate, and hence there is an urgent need for an in-depth analysis of various metabolic malfunctions that alter the proper functioning of a cell. Lipid metabolism is a process that involves the synthesis and simultaneous degradation of lipids and encompasses a balance that is essential to maintain the structural and functional ability of a cell. Androgen receptor (AR) plays a critical role in regulating cellular functions. Recent studies have expanded our knowledge regarding direct or indirect interactions that occur among mitochondria, peroxisome, and androgen receptors, which play a crucial role in lipid homeostasis. Unusual levels of lipids and cholesterol due to receptor excitation or inhibition are associated with multiple diseases and have been a cause of concern. The androgen receptor, along with other receptors and proteins, forms an important metabolic cascade that, if altered, may cause the accumulation of lipids and result in neurodegenerative disorders. In this review, we underscore the role of the androgen receptor in regulating lipid and cholesterol levels during neurodegenerative disorders (Alzheimers, Parkinson’s, multiple sclerosis, and Huntington’s disease).

**Main Points**

- Unusual levels of lipids and cholesterol due to androgen receptor excitation or inhibition are associated with neurodegenerative disorders in both males and females.
- The crosstalk of mitochondria and peroxisomes with androgen receptors is known to alter lipid homeostasis. Similarly, cluster of differentiation (CD) 36 receptor, liver X receptors, and other receptors, yet unknown, also play a vital role in understanding neurodegeneration.
- In this review, we underscore the role of the androgen receptor in regulating lipid and cholesterol levels during neurodegenerative disorders (Alzheimer’s disease, Parkinson’s disease, multiple sclerosis, and Huntington’s disease).

## Introduction

Androgen receptor (AR) is a known transcriptional activator of various pathways in a cell.^1^ The gene expression of AR initiates a cascade of reactions that stimulate other receptors and proteins present in a cell, thereby regulating metabolic functions. An alteration in these metabolic processes causes varied malfunctions. Lipid metabolic events form a core part of a chain of molecular events that are responsible for the proper functioning of a cell. The AR is known to affect the lipid cascade at multiple levels.^2,3^ Till date, some studies have shown the role of AR in regulating the expression of cellular genes and proteins. Previously, in a study on multiple sclerosis, AR was observed to adversely regulate transcription factors of members of the Transforming growth factor TGFF-β/SMAD pathway.^4^ TGF-β signaling is known to promote lipid accumulation while inducing lipogenesis-related genes and suppressing β-oxidation-related genes.^5^ In another study by Hung et al (2019), AR was shown to alter the expression of miRNA-204-5p to modulate the brain-derived neurotropic factor (BDNF) that influences depressive-like behavior in a chronic mild stress model.^6^ Mutations in the BDNF gene and its receptor, tyrosine receptor kinase B (TrkB), in the hippocampus have been reported to cause severe obesity in rodents.^7^ Even with these studies in place, the process by which AR regulates lipid metabolism is not yet elucidated. In this review, we underscore the role of the AR in regulating the lipid metabolic events during certain neurodegenerative disorders.

Neurodegeneration is an umbrella term for various neural disorders. Brain functioning is affected by AR signaling. A distribution map of AR showed widespread AR mRNA in neurons, specifically in regions involved in learning and memory such as the hippocampus and amygdala.^8^ Androgen receptors are also expressed in the hypothalamus, telencephalon, and amygdala and in the majority of the brain stem and spinal cord associated with sensory functions.^9^ At the cellular level, ARs are found on axons and dendrites, suggesting an essential and novel extra-nuclear role in neuronal functions.^10^ Androgens have been shown to exert an essential role in remyelination and axon regeneration, and testosterone has been shown to exert therapeutic potential in certain cases of neurodegeneration.^11^ Furthermore, loss of AR function leads to the breakdown of varied pathways affecting sensory as well as motor neuron functioning.^12^ The process of lipid intake and regulation of its levels within the cell is maintained by activation of the AR.^13^ Both the genomic and non-genomic activation of ARs helps in maintaining the cellular lipid homeostasis. Regular transcription of genes through translocation of AR to the nucleus falls under the genomic form of regulation. The non-genomic form of regulation by AR is one of the most interesting biological phenomena that have many facets which are yet undiscovered. To cite a study in relation to the non-genomic activity of AR, Benten et al (1997) studied that unconventional activation of calcium influx in T cells is the cause of plasma membrane receptors that are indirectly affected by testosterone-activating ARs.^14^ Most of the non-genomic studies todate have concentrated on abnormal AR stimulation, leading to cancer.^15^ Future clinical studies may well investigate androgen therapy as a promising avenue of treatment for demyelinating diseases and the aging process in both male and female patients.

Since the working of AR is still a mystery, and with the increasing prevalence of neural maladies, so analyzing and understanding the role of this particular receptor has become a necessity.

## Androgen Receptor and Other Receptor Interactions

**Figure 1.**
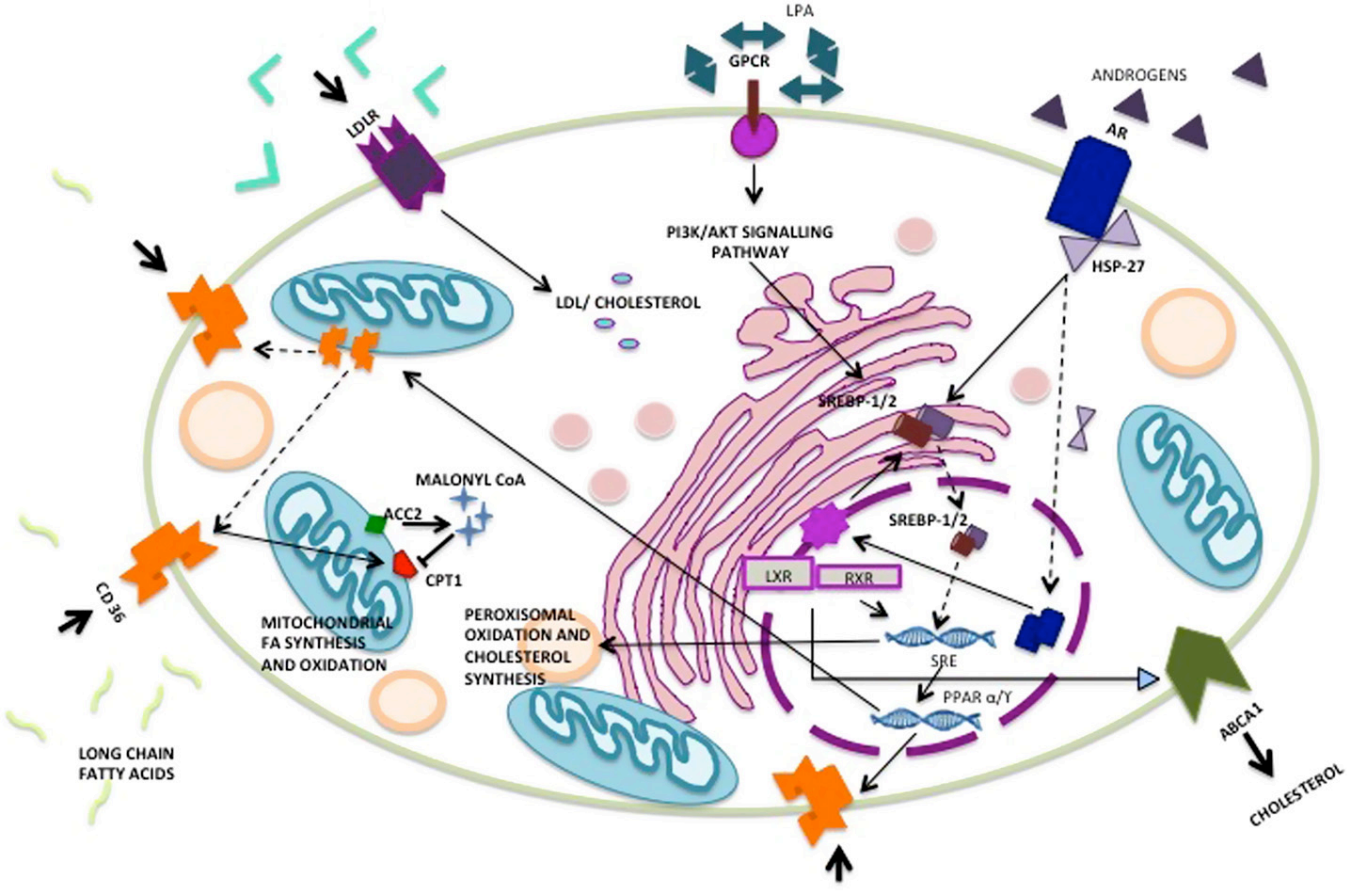
Various cell receptors and their mechanisms of action regulating lipid movement. Solid arrows depict the path of activation, whereas dashed arrows depict the movement of receptors [translocation] within the cell.

Androgen receptor is known to perform a central role in the metabolic functioning of a cell. The combination of ARs and liver X receptors (LXRs) regulates cholesterol levels and also performs lipid uptake in cells by activating the transcriptional factor Sterol regulatory element binding proteins SREBP^16^ (Figure 1). Studies have also shown the activation of peroxisome proliferator-activated receptor (PPAR) gamma^17^ by AR, which in turn regulates the activity of CD36,^18^ a known fatty acid transporter protein.

Deficiency in CD36 is known to cause dyslipidemia, subclinical inflammation, and metabolic disorders, which are established risk factors for atherosclerosis.^19^ Abnormally up-regulated CD36 also promotes inflammation, foam cell formation, endothelial apoptosis, macrophage trapping, and thrombosis.^20^ Any form of imbalance in CD36 levels alters the lipid uptake, and a study by Grajchen et al (2020)^21^ demonstrated that the lipid entry into the cell is dependent on the functioning of the NRF2 gene. While gene expressions of LXR-responsive, PPAR-responsive genes Abca1, Scd1, CPT1A, and Apoe are compromised following inhibition of CD36, it also plays an important part in activating the AMPK pathway to stimulate fatty acid oxidation, conducts intracellular signals, and activates inflammatory pathways such as Toll-like receptor,^22^ NF-κB, and c-Jun N-terminal kinase signals that control the cellular inflammatory response.^23^

As stated earlier, CD36 receptor translocation to the lipid raft or other nuclear receptors, including LXRs, is known to regulate lipid storage or oxidation. Liver X receptors have multiple endogenous ligands (Figure 1), several of which are oxysterol metabolites that have been demonstrated to be effective at physiological concentrations.^24^ In a study by Jung et al (2008), pharmacological activation of LXR was able to lower androgen activity by inducing enzymes essential for metabolic deactivation of androgens.^25^

In the central nervous system (CNS), LXRs act as lipid and cholesterol regulators, particularly when it comes to glial cell regeneration and myelin formation. Not just this, LXR has also been studied to perform a neuroprotective role by lowering neuroinflammation.^26^ Various neurodegenerative disorders like Amyotrophic lateral sclerosis ALS, Alzheimer’s disease (AD), and multiple sclerosis have been associated with dysregulation of cholesterol and oxysterol levels. Liver X receptor and AR have an antagonistic relation.^27^ The action of androgen and steroid hormones on the AR downregulates the activity of LXR.^16^ Other steroid hormone receptors, including estrogen, glucocorticoid, and progesterone receptors, may perform likewise. 

The mechanisms underlying non-classical signaling pathways remain elusive, though pathways such as Ras/Raf/MEK/ERK and PI3K/AKT/mTOR often display androgen-independent AR activation. The cross talk between AR and PI3K/AKT/mTOR as well as MAPK pathways has been extensively studied in cases of Polycystic ovary syndrome PCOS,^28^ prostate,^29^ and breast cancer,^30^ where such cross talks and reciprocal feedback loops with blockade of one lead to stimulation of the other and thus influence disease progression and recurrence. Further, while studying β-amyloid toxicity in AD, androgens were observed to reduce neuronal apoptosis by activating an intracellular AR-dependent non-genomic signaling cascade encompassing the activation of the MAPK/ERK pathway.^31^ Our knowledge on the association between multiple cellular receptors/genes and their trajectory of stimulation is still incomplete, and thus further studies are required to elucidate the pathways more comprehensively.

## Effect of Androgen Receptor on Mitochondria

There exists an inverse relationship between AR expression and mitochondrial DNA content, which has been demonstrated in a recent study.^32^ The AR is believed to translocate from the membrane to the nucleus, where activation of transcription factors takes place.^33^ It has been shown in an earlier study that AR localizes in mitochondria in prostate cells. AR contains a 36-amino-acid-long mitochondria localization signal that is capable of targeting the mitochondria and affects its regular functioning. In mitochondria, translation of mtDNA gene encoding is dependent on AR expression.^32^ The OXPHOS supercomplexes are destabilized in AR-expressing cells and have been restored upon AR knockdown.^32^ Oxidative stress is a natural process that intensifies with age, and a study on elderly mice revealed elevated levels of lipid peroxidation, decreased ratios of glutathione (GSH) to oxidized glutathione (GSSG), and reduced activity of mitochondrial complexes.^34^ Further, neurodegenerative diseases like AD and Parkinson’s disease (PD) are linked with age and may involve dysfunction of the AR in mitochondria that leads to an excessive generation of reactive oxygen species and hence results in oxidative stress.^35^ It is also known that AR signaling undergoes changes as we get older.^36^ Therefore, based on current knowledge, there appears to be a correlation between increased oxidative stress, lipid peroxidation, and AR signaling. However, the precise pathway linking these factors still needs to be explored.

Mitochondria produce biochemicals that act as biomarkers of neurodegenerative diseases. Cell growth and repair depend on the biosynthesis and breakdown of phospholipids, and to regulate such a course, the interplay of key biochemical events in both mitochondria and peroxisomes happens. Transcriptional nuclear genes like AR activate various pathways to undertake these lipid metabolic requirements. The Cytosine Adenine Guanine CAG repeats in muscular dystrophy is understood to be affected by AR disruption.^37^ The expansion of the AR gene causes abnormal nuclear accumulation of the AR protein, which initiates transcriptional dysregulation and subsequently causes axonal transport disruption, thereby disrupting mitochondrial functions.^38^ Androgen receptor protein with increased polyglutamine stretch has been seen to cause spinal and bulbar muscular atrophy. This particular neurodegenerative disorder shows similar lipid anomalies, which might lead us to a better understanding of the pathway involving the role of the AR.^39^ Mitochondria, being the powerhouse of the cell, acts upon the Peroxisome proliferator-activated receptor (PPAR) machinery to regulate cellular machinery. Androgen receptors and PPAR alpha are directly or indirectly involved in lipid metabolism.^40^ Thus, by correlating mitochondrial fatty acid beta oxidation and its association with PPAR alpha, the AR may act as a master regulator of cellular energy metabolism.

## Effect of Androgen Receptor on Peroxisomes

Plasmalogens produced in peroxisomes make up around 20% of the total phospholipid mass in humans, especially in brain, heart, and white blood cells.^41^ Peroxisomes are involved in the synthesis of dolichols, which are molecules present in cellular membranes and help in increasing membrane fluidity.^42^ Peroxisome proliferator-activat receptor gamma is necessary for adipogenesis.^43^ Just like PPAR gamma, ARs play a major role in activation of lipid synthesis. Peroxisome proliferator-activated receptor gamma with ligands as peroxisome-produced substrates is known to have a bidirectional interaction with AR. These receptors have been studied to influence the lipid levels and expression of genes within prostatic tissues. Not just in the prostate; such studies are being done in the brain, as well.^44^ A single peroxisomal gene lapse causing enzyme phytanoyl-CoA hydroxylase dysfunction has been known to result in adult Refsum’s disease.^45^ These oxidative enzymes are known to be target genes of PPAR alpha. Peroxisome proliferator-activated receptors are nuclear transcriptional regulators that indirectly interact with AR, and a lot is yet to be learned about the interplay.

## Lipid Metabolic Disorder in Neurodegenerative Diseases: Role of the Androgen Receptor

**Figure 2.**
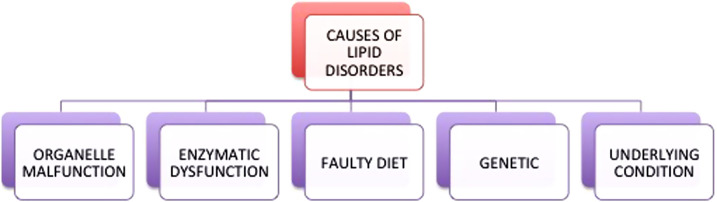
Causes of lipid disorders

Lipid metabolic malfunctions can either happen “because” of the disease or be a “cause” of the disease. A range of factors can cause lipid metabolic disorders, which include genetic disorders, faulty diets, or an underlying medical condition like diabetes, hypothyroidism, or even kidney disease (Figure 2). The basic test to determine any lipid abnormality can be performed by taking a blood sample to study the lipid profile. The disorders can be attributed to either organelle dysfunction or the faulty work of enzymes involved in lipid metabolism. Organelles that invoke the metabolism of lipids, as stated earlier, are mitochondria and peroxisomes. Disorders like Gaucher disease and Tay Sachs disease happen because of the low enzymatic activity of glucocerebrosidase and beta-hexosaminidase A, or the body is not able to convert fats into energy.^46^ Organelle-based lipid dysfunctions^47^ are more serious in nature, and the chances of survival in such cases are low, whereas in the case of enzyme-based lipid dysfunctions, enzyme replacement therapy can possibly be a way out Table 1.

Fatty acids are the building blocks of more complex lipids. Triglycerides are the storage form of fatty acids that get degraded via beta oxidation while releasing energy for ATP production.^48^ The brain is highly enriched in long-chain polyunsaturated fatty acids (LCPUFAS) docosahexaenoic acid (DHA) and arachidonic acid (AA).^49^ In the brain, polyunsaturated fatty acids (PUFA) are dominant precursors for the biosynthesis of lipid mediators, which activate the inflammatory response.^50^ Free fatty acids, especially those present in the cortical regions, when elevated, induce protein aggregation, whereas long-chain fatty acids enhance assimilation to some extent.^51^ Cellular metabolic changes within the brains of people with neurodegenerative disorders are seen very early and affect multiple metabolic pathways.^52^ There are multiple evidences of altered mitochondrial and peroxisomal functions in AD, PD, ALS, and many other such neural disorders, which are discussed below.

### Alzheimer’s Disease

Alzheimer’s disease is the most common disease of the elderly, and its pathology needs to be understood and addressed in view of it being a public health problem. In the brain, 2 fatty acids are known to be present in abundance: AA and DHA.^53^ Cellular metabolic changes within the brain of people with AD are seen very early and precede the development of both amyloid plaques and neurofibrillary tangles.^54^ In AD, an increased amount of lipid peroxidation has been seen with decreased levels of polyunsaturated fatty acids.^55^ Also, membrane fluidity has been shown to decrease with an increased amount of hydroxynonenal, a free radical second messenger, and a neurotoxic aldehyde of polyunsaturated fatty acid oxidation.^56^ Further, deficits in the function of mitochondria, especially oxidative phosphorylation and the lipid breakdown machinery, leads to the accumulation of excess reactive PUFA in the cell, which causes fibrillation and plaque formation.^57^

Mitochondrial functioning is also affected by unregulated levels of apoenzyme isomers, which are seen to be altered in case of AD.^58^ Apoenzyme levels are associated with the severity of AD pathology. The isoform e4 genotype is seen to influence severity of both axonal tau phosphorylation and amyloid-induced neurite pathology.^59^ Altered AR functioning is seen in AD with a probable interaction between apoenzyme isomer 4 and AR, which is yet to be fully understood. These anomalies are seen to normalize following the administration of testosterone.^60^

Increased levels of very long-chain fatty acid accumulates are found in the cell to determine peroxisomal dysfunction.^61^ The increased very long chain fatty acid (VLCFA) levels are associated with the presence of neurofibrillary tangles.^62^ Not just the VLCFA but also hypercholesterolemia is closely related to AD,^63^ though the exact mechanism is unknown. DHA, being a major determinant of neural health and development, is seen to be present in decreasing levels.^64^ Exogenous introduction of DHA has been studied to increase neural levels, showing anti-inflammatory effects by increasing blood flow and thus decreasing amyloid aggregation in the brain.^65^ Alzheimer’s pathological characteristics depict impaired plasmalogen biosynthesis,^66^ which is so far unresolved. Plasmalogen deficiency is known to aggravate the disease symptoms, amplifying AD in the brain.^67^ Just like plasmalogens, peroxisomal disruption alters ether lipid levels.^61^ A study on physiological changes that take place in ether-lipid-deficient mice showed disturbed androgen-dependent regulation.^68^ In the case of AD, abnormal ether lipid levels have also been studied,^69^ but the exact role of AR in regulating plasmalogens and ether lipids is yet to be consolidated.

There is a popular debate on the gender-specific early onset of AD. Women and men both develop AD mostly at a later stage in life. However, in a study by Podcasy et al (2016),^70^ it is seen that women are at a 2-old risk of developing late AD compared to men. The increase in susceptibility is majorly due to underlying genetic causes. The human AR is a transcription factor that is located on the X chromosome. The chromosome activation and inactivation at random positions make a female vulnerable to sex bias that may result in a specific phenotypic effect.

### Parkinson’s Disease

Parkinson’s disease is a neurodegenerative disorder characterized by aggregation of Lewy bodies and accumulation of alpha-synuclein. Parkinson’s disease is the second most common neural disorder and involves dysregulation of polyunsaturated fatty acids, which are critical for neuronal membranes to maintain cell membrane fluidity and permeability.^71^ Not just in the structural arena, these fatty acid components take part in intracellular and extracellular signaling as second messengers and also act as a reservoir of energy when in need.^72^ The interaction of alpha-synuclein with either oxidized or non-oxidized PUFAs could lead to mitochondrial changes.^73^ The genes PLA2G6 and SCARB2 following AR activation are involved in lipid metabolism and have been reported to be associated with PD.^74^ Parkin, DJ-1, PINK1, and LRKK2 are common proteins that are implicated in the regulation of mitochondrial functions.^75^ Mutations in these proteins could lead to inherited Parkinson’s disease.^76^ Pathological modifications in the substantia niagra, pars reticulata, and GABAA-ergic circuit are evident during Parkinson’s. Testosterone circulation is seen to play a protective role in restoring motor behavior in these regions of the brain.^77^ Important sites of testosterone have been determined to be in the pelvic autonomic ganglion cells. These cells have a relation with autonomic reflex function, which is seen disturbed in Parkinson’s.^78^ In a study by Alam and Schmidt (2004),^79^ androgen levels are seen to influence dopaminergic function via an increase in tyrosine hydroxylase mRNA expression. Thus, the AR-mediated metabolic pathway is evident in the case of Parkinson’s.

Similar to AD, patients of Parkinson’s show altered levels of DHA^80^ and arachidonic acid,^81^ whereas saturated fatty acids are enhanced in the disease.^82^ However, unusually lower levels of cholesterol and triacylglycerols are seen in Parkinson’s, including decreased levels of ethanomlamine plasmogens.^83^ Peroxisome proliferator-activated receptor modulation has been studied to check the activity of dopaminergic neurons. Peroxisome proliferator-activated receptor gamma is a known isomer that is modulated by AR activation.^84^ In Parkinson’s disease, reduced levels of plasmalogens in the lipid raft have been studied. Not just plasmalogens, but similar to AD, reduced lipid ethers have also been seen in the cortical gray matter of PD patients.^85^ Androgenic receptor interplay with the peroxisome might be a way to alter lipid metabolism anomalies in PD. Further studies are required to understand the real cause of varied lipid metabolic malfunctions in the peroxisomes.

Sex bias studies have shown that men are 1.5 times more prone to the early onset of PD as compared to women.^86^ The difference in onset between men and women has been noted to be 2 years. The decrease in progression in women is mostly due to high levels of estrogen before menopause. The ovarian hormones might act as a protective factor in women against PD.^87^ Men with normal aging are seen to decrease the levels of testosterone at a progressive rate in their 50s.^88^ The deficiency might be the cause of the increased prevalence of PD in men. Not just the androgen levels but also the AR levels get altered during old age, and hence a cause of PD. The exact relationship between the level of androgen and AR in a cell with the progression of PD is still a mystery. However, androgen inhibitors have shown a neuroprotective role in the presence of testosterone. Therefore, more studies are required to elucidate the exact mechanism and cause of the higher prevalence of PD and the role of AR in men.

### Multiple Sclerosis

This particular disease is characterized by impairment in myelin formation. Damage to the myelin sheath in turn causes damage to the neurons, making them vulnerable to stimuli causing speech impairment, a lack of muscular coordination, etc. The process of myelin regeneration is a major therapeutic goal in demyelinating diseases, and the failure to remyelinate rapidly has profound consequences for the health of axons and for brain function. However, no efficient treatment has been formulated till date except the use of anti-inflammatory drugs.^89^ It has been noticed that males are less likely to develop multiple sclerosis but often develop a severe disease course, with aggressive symptoms appearing at an earlier age as compared to females.^90^ The activated astrocytes and microglial cells increase considerably during the course of the disease, and it has been seen that on testosterone administration, the repair mechanism could be triggered and the number of astrocytes and microglial cells may return to control levels.^91^ It may well be argued that AR can be used as a probable target, and drugs may be delivered specifically to alter the disease conditions and myelin recovery. Androgen receptor at the neural level is necessary for remyelination, and in a paper by Hussain et al (2013),^92^ AR present in microglia and peripheral tissue was able to activate and enhance remyelination in the presence of testosterone.

### Huntington’s Disease

Huntington’s disease is an autosomal dominant disorder that runs in a family. A progressive neurodegenerative disorder, Huntington’s disease, causes uncontrolled movements known as chorea that involve involuntary jerking or twitching movements.^93^ The patients also show dominant emotional and behavioral alterations.^94^ This disease is known to be caused by an increase in the CAG segment repeat (around 35-120) in the HTT gene, which forms the huntingtin protein. The people with 40 or more repeats show evident symptoms of Huntington’s disease. The role of normal huntingtin protein is yet elusive to us but is studied to be of importance in neurons.^95^ The AR in neurons on mutation has been correlated with a diverse range of maladies, including androgen insensitivity, spinal bulbar muscular atrophy, and neuromuscular degeneration.^96^ The ployglutamine repeats within the N terminal of the receptor is the major cause of these malfunctions.^97^ Therefore, studying the AR as a probable Huntington’s disease associate is the need of the hour and will help in extending our knowledge about how amino acid repetitions play a role in aggravating the diseased conditions.

## Conclusion

Androgen receptors, on either activation or inhibition, regulate lipid homeostasis in the cell. These receptors can be modulated to alter the progression of neurodegenerative diseases. Androgen receptors in congruence with CD36, LXR, and multiple other receptors, yet unknown, play a vital role. As explained, ARs are mostly available in the hippocampus region, which is a hub of metabolic processes when it comes to learning and memory. With continuous reshaping of synapse and high metabolic needs, both lipid biosynthesis and oxidation cycles are highly active de novo. Also, studies on neural lipid metabolic disorders have shown that testosterone can activate AR and show evidence of a potential neuroprotective role in AD. Thus, for future therapeutic regimens, AR is an excellent target; however, efficient penetration of the AR antagonist through the blood–brain barrier is obligatory for the treatment of brain maladies.

With more studies being done on crosstalk of organelles, the interaction between mitochondria and peroxisome is seen, especially in cases of peroxisomal disorders, where both are affected equally. The studies, as discussed earlier, show that the lipid metabolic pathway is disrupted in neurodegenerative diseases. The lipid anomaly multiplies interfering with protein folding assisting in tissue damage, making the pathology of the disease visible. Also, the transcription factors of the PPAR type are the key regulators of peroxisomal fatty acid beta-oxidation, with PGC-1 alpha regulating the mitochondrial biogenesis. However, PPARs not only affect lipid metabolic pathways but are also involved in varied other metabolic functions in the cell. These molecules are major interlinks that get altered in almost every disease, and hence, using AR to modulate PPAR isomers at the transcriptional level may act as an attractive target, thereby altering multiple signaling pathways that can go either way. With all this in mind, we still have not been able to elucidate the exact proteins that interact during lipid exchange and how these defects in peroxisomes or mitochondria influence the metabolic alterations during diseased conditions.

A lot of independent studies on lipid synthesis and oxidation in neurodegenerative disorders have been established in multiple labs. To fill up the loopholes in receptor interactions and lipid metabolic trajectory at various organelle levels, much is yet to be studied. This review will help in extracting multiple new receptors and proteins as targets for drug therapy as well as help in better understanding AR as a target against lipid metabolic disorders in neurodegenerative diseases.

## Figures and Tables

**Table 1.** Lipid Malfunction in Various Neurodegenerative Disorders with their Pathological Characteristics^52^

S.no	Neurodegenerative Disease	Pathology	Lipid Malfunction
1.	Alzheimer’s	Beta amyloid aggregates + hyperphosphorylated tau protein.	Decrease in docosahexaenoic acid levels and increase in arachidonic acid, elevated triglyceride and diacylglycerol with altered cholesterol levels.
2	Parkinson’s	Alpha synuclein protein, dopaminergic neuron damage.	Peroxidation of lipids rich accelerates the metabolism of dopamine. Significant increase in unsaturated fatty acids.
3	Amyotrophic lateral sclerosis.	Collapse of large pyramidal neurons, glutamate toxicity.	Accelerated fat loss in adipocytes, modulation in glycosphingolipids, and SOD1 aggregation.
4	Multiple sclerosis.	T lymphocyte-mediated disorder. Increased pentane and ethane in urine.	Excess degradation of unsaturated fatty acids. Thiobarbituric acid reactive substances and F-isoprostane levels elevated in CSF (Cerebrospinal Fluid).
5	Huntington’s.	CAG(cytosine,adenine,guanine) repeats - polyglutamine tract.	Impaired cholesterol metabolism. MHTT also curtails maturation of SREBP, upregulating LXR genes. Insulin resistance seen too.
